# Retinal Arteriosclerosis in a Large Health Screening Cohort: Associations with Systemic Vascular Comorbidities and Stroke in Young Adults

**DOI:** 10.3390/biomedicines14051035

**Published:** 2026-05-02

**Authors:** Kunho Bae, Ju-Yeun Lee, Hyuk Jin Choi

**Affiliations:** 1 Department of Ophthalmology, Seoul National University Hospital, Seoul 03080, Republic of Korea; luben81@gmail.com; 2 Mass Eye and Ear and the Department of Ophthalmology, Harvard Medical School, Boston, MA 02114, USA; 3 Department of Preventive Medicine, Seoul National University College of Medicine, Seoul 030380, Republic of Korea; leejy5293@gmail.com; 4 Biomedical Research Institute, Seoul National University Hospital, Seoul 03080, Republic of Korea; 5 Department of Ophthalmology, Boston Children’s Hospital, Boston, MA 02115, USA; 6 Department of Ophthalmology, Seoul National University Hospital Healthcare System Gangnam Center, Seoul 06236, Republic of Korea; 7 Department of Ophthalmology, Seoul National University College of Medicine, Seoul 03080, Republic of Korea

**Keywords:** arteriosclerosis, fundus photography, hypertension, metabolic syndrome, retinal vessels, stroke

## Abstract

**Background:** Routine fundus photography is widely accessible; however, its utility in stratifying systemic vascular risk in asymptomatic, general populations remains understudied. We utilized a large-scale health screening cohort in South Korea to evaluate the clinical validity of the retinal arteriosclerosis index (RAI) in a generally healthy population. **Methods:** We conducted a cross-sectional study of 74,608 adults who underwent routine health screening (2003–2010) at a tertiary center. Retinal arteriosclerosis was graded (0–4) by masked readers with a modified Scheie classification; a higher eye grade was defined as a person-level grade. High-grade RAI was prespecified as ≥2. Associations with systemic conditions (hypertension, type 2 diabetes, hyperlipidemia, metabolic syndrome, cardiovascular disease, and stroke) were examined by using multivariable logistic regression adjusted for demographic, lifestyle, and laboratory covariates; moreover, analyses were stratified by age and sex. **Results:** High-grade RAI was present in 4.5% of the participants and increased with age. After adjustment, high-grade RAI was associated with hypertension (OR, 2.97; 95% CI, 2.73–3.23), diabetes mellitus (OR, 1.35; 95% CI, 1.22–1.50), cardiovascular disease (OR, 1.46; 95% CI, 1.25–1.71), metabolic syndrome (OR, 1.63; 95% CI, 1.49–1.78), and stroke (OR, 1.98; 95% CI, 1.41–2.79) but not with hyperlipidemia. Sex-stratified analyses revealed broadly similar patterns, although high-grade RAI was associated with stroke in women and cardiovascular disease in men. Age-stratified analyses demonstrated consistent associations with hypertension, metabolic syndrome, and stroke across all age groups, with stronger effect sizes being observed in younger individuals. With respect to lifestyle factors, frequent alcohol consumption was associated with higher odds of high-grade RAI. Laboratory correlates included higher uric acid levels and lower red blood cell, albumin, and bilirubin levels (all *p* < 0.001). **Conclusions:** Fundus-defined arteriosclerotic changes were independently associated with multiple systemic vascular and metabolic conditions. An association with stroke in adults younger than 40 years of age was also observed, although this finding should be interpreted with caution given the cross-sectional design and limited number of events. These findings support the potential role of retinal vascular changes as cross-sectional correlates of systemic vascular health. Longitudinal studies are needed to clarify temporal relationships and causality.

## 1. Introduction

Retinal vascular signs captured on fundus photographs have long been investigated as surrogate markers of systemic vascular health [1,2,3,4,5]. These signs reflect structural remodeling of the arteriolar walls driven by chronic hemodynamic stress and metabolic dysregulation [6,7,8,9,10]. Previous large-scale prospective cohort studies, including the Atherosclerosis Risk in Communities (ARIC) study, have demonstrated that retinal microvascular abnormalities are associated with incident cardiovascular and cerebrovascular events [11]. However, these landmark studies have largely focused on middle-aged or older populations with established vascular risk profiles. Therefore, the clinical significance of retinal arteriosclerosis in the generally healthy, younger working-age population remains largely unexplored.

In this context, the health screening program in South Korea offers a unique opportunity to address this research gap. Facilitated by high medical accessibility, the widespread availability of screening centers for the general public provides a robust database for medical research. Unlike hospital-based registries that are often biased toward individuals with established diseases, these screening cohorts generate standardized fundus photography data from a broad, largely asymptomatic adult population [12]. This real-world dataset enables the detection of subclinical retinal vascular changes before overt systemic disease becomes clinically apparent, thus providing clearer insights into the early relationship between retinal morphology and systemic health [13].

In this study, we applied a modified retinal arteriosclerosis index (RAI) grading system (a fundus-based scale reflecting the retinal arteriolar light reflex and vascular narrowing) to a large health screening cohort of 74,608 individuals [8]. Rather than focusing solely on known associations, our primary objective was to examine whether fundus findings are associated with systemic vascular conditions at the population level. In addition, we explored the relationships between the RAI and laboratory parameters, as well as modifiable lifestyle factors, to assess its potential relevance as a marker of vascular status and cumulative behavioral and metabolic exposure.

## 2. Methods

This population-based cross-sectional study was approved by the Institutional Review Board of Seoul National University Hospital (IRB No. H-1906-141-1043). The requirement for informed consent was waived because of the retrospective nature of the study and the use of deidentified data. All of the study procedures adhered to the tenets of the Declaration of Helsinki.

### 2.1. Study Design and Participants

We analyzed data from individuals who underwent comprehensive health screening at the Seoul National University Hospital Healthcare System Gangnam Center, which is a tertiary screening facility affiliated with the Healthcare Research Institute. All consecutive individuals aged 20 years and older who underwent comprehensive health screening (including fundus photography) between October 2003 and December 2010 were included. Nonmydriatic fundus photographs with a 45° field angle were acquired by using a digital fundus camera (CR6-45NW; Canon Inc., Tokyo, Japan) in a dark room. Eyes with poor-quality photographs that precluded reliable grading, such as those with severe media opacity (e.g., dense cataracts) or poor image focus, were excluded. In addition, eyes with degenerative myopia exhibiting extensive peripapillary atrophy that hindered reliable assessments of retinal arteriosclerosis were also excluded. Additional data collected during the screening process included anthropometric measurements, survey-based lifestyle information, past medical history, and laboratory test results. No a priori sample-size calculation was performed; moreover, we included all consecutive eligible participants during the study period.

### 2.2. Retinal Arteriosclerosis Grading (Procedures)

**Figure 1:**
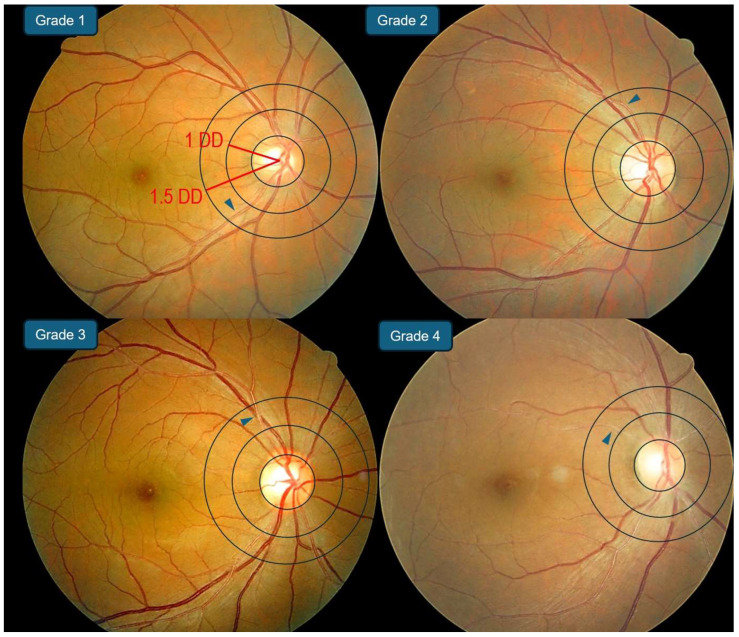
Representative fundus photographs illustrating the retinal arteriosclerosis index (RAI) grading system, adapted from the Scheie classification. Grading was focused on retinal arterioles located within 1.0 to 1.5 disk diameters from the center of the optic disk. Blue triangles indicate representative sites of retinal arteriosclerosis.

Fundus photographs were independently reviewed by two trained graders (K.B. and H.J.C.) who were blinded to the participants’ clinical information and the chronological order of the photographs. Disagreements were adjudicated via discussion with a third specialist (J.L.). Retinal arteriosclerosis was evaluated by using the RAI, which is based on the Scheie classification (with slight modifications) and comprises five levels of arteriolar change (Figure 1) [8,14,15]. Grade 0 indicates no apparent change; grade 1 represents barely detectable arteriolar narrowing with widening of the light reflex and minimal or no AV compression; grade 2 (mild) is defined as obvious arteriolar narrowing with focal irregularities, a prominent light reflex, and the presence of AV crossing changes; grade 3 (moderate) corresponds to a “copper-wire” appearance of the arteries with more pronounced AV crossing signs; and grade 4 (severe) is defined as a “silver-wire” appearance with the most severe AV crossing changes.

Grading focused on arterioles located within 1.0–1.5 disk diameters from the center of the optic disk by using standardized reference photographs. For analysis, individuals were categorized into three groups: normal (grades 0–1), mild (grade 2), and marked (grades 3–4). Each eye was independently graded, and the higher eye grade defined the person-level grade. For dichotomous classification, high-grade RAI was prespecified as ≥2.

Grade 0 indicates no apparent arteriolar change.

Grade 1 denotes barely perceptible arteriolar narrowing with widening of the light reflex.

Grade 2 (mild) indicates obvious arteriolar narrowing with focal irregularities, a prominent light reflex, and the presence of arteriovenous (AV) crossing signs.

Grade 3 (moderate) corresponds to a “copper wiring” manifestation, which is characterized by increased vessel wall opacity with partial visibility of the blood column and prominent AV crossing signs.

Grade 4 (severe) corresponds to a “silver wiring” manifestation, with complete vessel wall opacification, loss of blood column visibility, and severe AV crossing signs.

### 2.3. Clinical Data from Standardized Health Screening Records

Data were extracted from standardized health screening records. Demographic information included age and sex. Lifestyle factors were assessed via structured questionnaires and included household income (categorized into quartiles), smoking status (never, former, or current), alcohol consumption (none, mild-moderate [1–4 times/week], or severe [≥5 times/week]), and physical activity (inactive [0–1 times/week] or moderate [≥2 times/week]).

Medical history was based on physician interviews and documented diagnoses, including hypertension, diabetes mellitus (DM), cardiovascular disease, cerebrovascular stroke, and metabolic syndrome. To ensure consistency, systemic diseases were defined using a combination of self-reported history, medication use, and laboratory criteria.

Participants were considered to have DM if they (1) reported a physician diagnosis of DM, (2) were using antidiabetic medications, (3) exhibited a fasting plasma glucose concentration ≥ 126 mg/dL, or (4) exhibited a glycated hemoglobin (HbA1c) level ≥ 6.5%. Hypertension was defined as (1) a self-reported diagnosis of HTN, (2) the use of antihypertensive medication, or (3) a measured systolic blood pressure (SBP) ≥ 140 mmHg and/or diastolic blood pressure (DBP) ≥ 90 mmHg. Hyperlipidemia was defined as (1) a self-reported diagnosis or use of lipid-lowering medication or (2) total cholesterol ≥ 240 mg/dL, triglycerides ≥ 200 mg/dL, low-density lipoprotein (LDL) cholesterol ≥ 160 mg/dL, or high-density lipoprotein (HDL) cholesterol < 40 mg/dL. Metabolic syndrome was defined according to the modified National Cholesterol Education Program Adult Treatment Panel III (NCEP ATP III) criteria for Asian populations [16]. Cardiovascular disease and cerebrovascular stroke were defined according to physician-reported history and/or documented medical records.

Anthropometric data, including body mass index (BMI) and blood pressure, were measured by trained healthcare personnel following standardized protocols. Laboratory tests included fasting glucose levels, HbA1c levels, complete blood cell counts, lipid profiles, renal function markers, inflammatory markers, liver function markers and protein markers.

### 2.4. Outcomes

The primary outcome of this study was the presence of systemic comorbidities according to the RAI grade. Specifically, the analysis focused on the associations between the high-RAI grade group (grade ≥ 2) and physician-diagnosed comorbidities, including HTN, DM, hyperlipidemia, metabolic syndrome, cardiovascular disease, and cerebrovascular stroke. Secondary outcomes included the associations between the RAI grade and (1) lifestyle-related factors (e.g., smoking, alcohol consumption, and physical activity), (2) anthropometric measurements (e.g., BMI and blood pressure), and (3) laboratory biomarkers reflecting metabolic and vascular health.

### 2.5. Statistical Analysis

All of the statistical analyses were performed by using SAS software (version 9.4; SAS Institute Inc., Cary, NC, USA). Continuous variables are presented as the means ± standard deviations or medians with interquartile ranges (depending on their distribution), whereas categorical variables are summarized as frequencies and percentages. The normality of the continuous variables was assessed prior to analysis, and appropriate parametric or nonparametric tests were applied accordingly. Continuous variables were compared by using Student’s *t* test or one-way analysis of variance (ANOVA), and the Kruskal–Wallis test was used when normality assumptions were not met. Categorical variables were compared by using the Pearson’s chi-square test.

Missing data were handled by using an available-case analysis approach without imputation. In the descriptive analyses, percentages were calculated based on valid responses, and the number of missing observations for each relevant variable was explicitly reported. For multivariable analyses, standard listwise deletion was applied within each model such that participants were excluded only if they had missing values for the variables included in that specific model.

Intergrader reliability was assessed using the quadratically weighted Cohen’s kappa statistic. Graded associations across RAI categories were evaluated by calculating adjusted *p*-for-trend values using multivariable logistic regression models. In these models, RAI grade was modeled as an ordinal variable (0 for Grades 0–1, 1 for Grade 2, and 2 for Grades 3–4), using the same outcome-specific covariate sets as in the final multivariable models.

Associations between the RAI and systemic comorbidities were evaluated by using multivariable logistic regression models adjusted for demographic, lifestyle, and selected clinical variables (including age, sex, body mass index, smoking status, alcohol intake, physical activity, household income, and relevant laboratory measures). To avoid overadjustment and circular modeling, variables overlapping with the diagnostic components of each outcome (e.g., systolic and diastolic blood pressure for hypertension) were excluded from the corresponding models. The results are reported as adjusted odds ratios (ORs) with 95% confidence intervals (CIs). For secondary outcomes, multivariable logistic or linear regression models were used to assess associations between the RAI and lifestyle factors, anthropometric measurements, socioeconomic variables, and laboratory biomarkers. In each analysis, variables treated as outcomes were excluded from the corresponding covariate set to avoid self-adjustment. Continuous biomarkers were standardized per 1 standard deviation increase before modeling. Subgroup analyses stratified by sex and age group were conducted to assess potential effect modifications. For the subgroup analysis of stroke in participants younger than 40 years, Firth’s penalized logistic regression was applied to address sparse data bias. A two-sided *p* value <0.05 was considered to indicate statistical significance.

## 3. Results

### 3.1. Study Population

**Figure 2:**
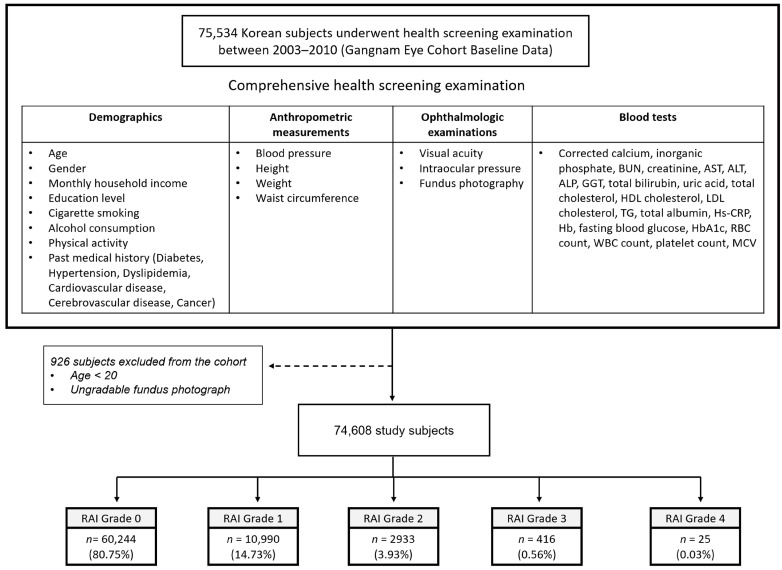
Flowchart of study subject selection and distribution of retinal arteriosclerosis index (RAI) grades. Among the 75,534 Korean individuals who underwent comprehensive health screening, 926 were excluded because they were aged <20 years or had ungradable fundus photographs, thus resulting in 74,608 participants for the final analysis.

Among the 75,534 Korean individuals who underwent comprehensive health screening, a total of 926 individuals were excluded because they were aged <20 years or had ungradable fundus photographs, thus resulting in 74,608 individuals for the final analysis (Figure 2). Using the RAI, 60,244 (80.75%) subjects were classified as grade 0, 10,990 (14.73%) were classified as grade 1, 2933 (3.93%) were classified as grade 2, 416 (0.56%) were classified as grade 3, and 25 (0.03%) were classified as grade 4. Overall, 3374 participants (4.52%) were classified into the high-RAI-grade group (those with grade ≥ 2). The intergrader agreement for the RAI grading was excellent (quadratically weighted Cohen’s κ = 0.93).

The prevalence of high RAI grades progressively increased with age: 0.6% in participants aged <40 years, 2.6% in the 40–49 years age group, 6.2% in the 50–59 years age group, 11.2% in the 60–69 years age group, and 12.5% in those aged ≥70 years. The baseline characteristics of the study population are summarized in Table 1. Individuals with high RAI grades were generally older, particularly those in the marked RAI subgroup. No significant sex differences were observed in the overall distribution of RAI grades.

### 3.2. Associations Between RAI and Systemic Comorbidities

Systemic comorbidities were more prevalent in individuals with higher RAI grades than in those in the control group (Table 1). The prevalences of hypertension, diabetes mellitus, metabolic syndrome, cardiovascular disease, and stroke increased with increasing RAI grades, whereas hyperlipidemia exhibited a less consistent pattern. Smoking and alcohol consumption appeared less frequently among individuals with higher RAI grades; this pattern was largely attributable to age-related confounding, as older participants in this cohort demonstrated lower rates of these behaviors.

In multivariable-adjusted models, clear graded associations were primarily observed for hypertension and stroke (Table 2). Compared with participants with RAI grades 0–1, adjusted odds ratios increased with higher RAI severity, particularly for hypertension (OR, 2.83; 95% CI, 2.59–3.10 for grade 2; OR, 4.23; 95% CI, 3.31–5.40 for grades 3–4) and stroke (OR, 1.76; 95% CI, 1.20–2.57 for grade 2; OR, 3.35; 95% CI, 1.74–6.45 for grades 3–4). Similar graded associations were observed for diabetes mellitus and metabolic syndrome (both *p*-for-trend < 0.001). While most outcomes showed significant *p*-for-trend values, the associations for hyperlipidemia and cardiovascular disease did not follow a clear monotonic pattern. Specifically, for cardiovascular disease, the association was most pronounced in grade 2 (OR, 1.52; 95% CI, 1.29–1.79) but attenuated and was no longer statistically significant in grades 3–4 (OR, 1.14; 95% CI, 0.73–1.77). Consistently, hyperlipidemia did not demonstrate a significant trend after full adjustment (*p*-for-trend = 0.541).

According to the multivariable models, high-grade RAI was independently associated with hypertension (OR, 2.97; 95% CI, 2.73–3.23), diabetes mellitus (OR, 1.35; 95% CI, 1.22–1.50), cardiovascular disease (OR, 1.46; 95% CI, 1.25–1.71), metabolic syndrome (OR, 1.63; 95% CI, 1.49–1.78), and stroke (OR, 1.98; 95% CI, 1.41–2.79) but not with hyperlipidemia (OR, 0.99; 95% CI, 0.91–1.08) (Table 3).

According to sex-stratified analyses, the overall pattern of the associations was broadly similar between men and women; however, the association with stroke appeared to be stronger in women (OR, 3.46; 95% CI, 2.08–5.78), whereas the association with cardiovascular disease was more pronounced in men (OR, 1.53; 95% CI, 1.27–1.85) (Table 3).

In the age-stratified analyses, high-grade RAI was associated with hypertension and metabolic syndrome across all of the age groups, with stronger effect sizes being observed in younger individuals. Notably, among participants younger than 40 years, high-grade RAI was associated with higher odds of stroke (OR, 21.25; 95% CI, 2.22–97.14). Given the limited number of stroke events in this subgroup (n = 10; 9 in the control group and 1 in the high-grade RAI group), this association was estimated using Firth’s penalized logistic regression. To minimize overfitting, the model was adjusted using a parsimonious set of covariates, including sex, body mass index, and smoking status.

### 3.3. Lifestyle Factors and the RAI

**Figure 3:**
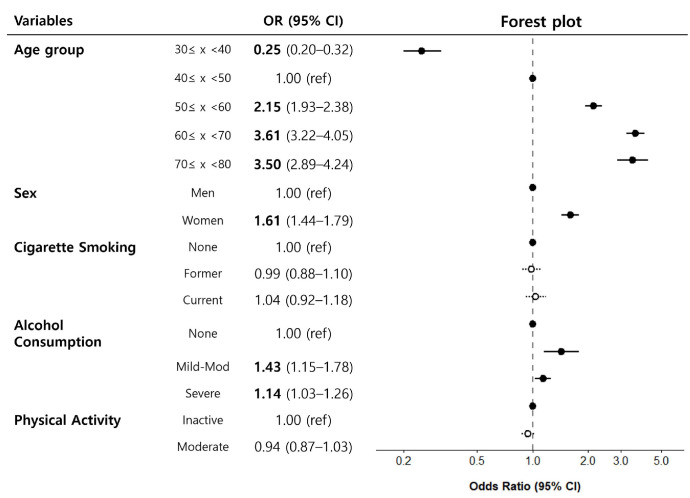
Lifestyle factors associated with a high retinal arteriosclerosis index (RAI) grade. A forest plot presents the associations between various lifestyle factors and high RAI grades. Adjusted odds ratios (ORs) with 95% confidence intervals (CIs) are shown for each factor. Black circles indicate statistically significant estimates (excluding the reference), whereas white circles indicate non-significant estimates. Statistically significant parameters are highlighted in bold.

High-frequency alcohol consumption was associated with increased odds of having a high RAI grade (Figure 3). In contrast, no consistent associations were observed for physical activity or smoking status after adjustments for potential confounders, including age, sex, and BMI.

### 3.4. Anthropometric Measures and the RAI

Participants with higher RAI grades exhibited significantly higher DBP (OR, 1.687; 95% CI, 1.620–1.758) and SBP (OR, 1.545; 95% CI, 1.487–1.606). Glycemic measures, including fasting glucose (OR, 1.134; 95% CI, 1.101–1.169) and HbA1c levels (OR, 1.111; 95% CI, 1.078–1.145), were also positively associated with higher RAI grades. BMI demonstrated a modest positive association (OR, 1.193; 95% CI, 1.144–1.244).

**Figure 4:**
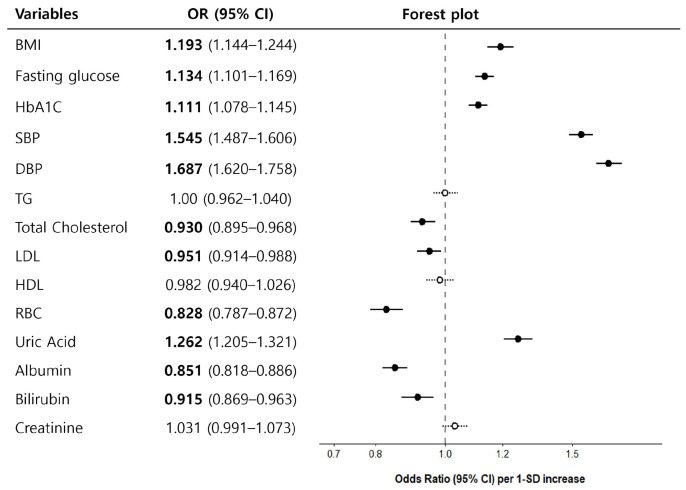
Body measurements and blood test results associated with a high retinal arteriosclerosis index (RAI) grade. Forest plot presenting the associations between various body measurements and blood test parameters and high RAI grades. Adjusted odds ratios (ORs) with 95% confidence intervals (CIs) are displayed for each variable. Black circles indicate statistically significant estimates (excluding the reference), whereas white circles indicate non-significant estimates. Statistically significant parameters are highlighted in bold.

In contrast, lipid-related measures demonstrated weaker and inconsistent associations, with total cholesterol (OR, 0.930; 95% CI, 0.895–0.968) and LDL cholesterol (OR, 0.951; 95% CI, 0.914–0.988) demonstrating modest inverse associations, whereas triglycerides and HDL cholesterol were not significantly associated. Adjusted estimates are summarized in Figure 4.

### 3.5. Laboratory Biomarkers and the RAI

In multivariable models adjusted for potential confounders, higher serum uric acid levels were positively associated with high-grade RAI (OR, 1.26; 95% CI, 1.21–1.32; *p* < 0.001), whereas lower red blood cell (RBC) counts, albumin levels, and bilirubin levels were inversely associated (RBC: OR, 0.83; 95% CI, 0.79–0.87; albumin: OR, 0.85; 95% CI, 0.82–0.89; bilirubin: OR, 0.92; 95% CI, 0.87–0.96; all *p* < 0.001). Adjusted estimates are summarized in Figure 4.

## 4. Discussion

In this large-scale, population-based study of 74,608 asymptomatic adults undergoing routine health screening, we reported that a high RAI grade was significantly associated with a higher prevalence of systemic vascular and metabolic comorbidities. The overall prevalence of high RAI grades was 4.5%, which clearly increased with age. Notably, higher alcohol consumption was independently associated with high RAI grades, whereas smoking and physical activity were not significantly associated after multivariable adjustment. In addition, we observed an association between retinal arteriosclerosis and stroke in individuals younger than 40 years. Although the confidence interval was wide (reflecting the small number of stroke events in this subgroup), this observation may reflect early vascular changes that are detectable by retinal imaging. In this context, retinal arteriosclerotic findings in younger individuals may represent an ophthalmoscopic correlate of early vascular risk (rather than merely incidental findings).

The biological plausibility of our findings may be explained by the distinct pathophysiology of retinal microvessels. In our multivariable analysis, high-grade RAI remained associated with hypertension and diabetes mellitus but not with hyperlipidemia. Systolic and diastolic blood pressures demonstrated particularly strong associations among continuous clinical variables, which are consistent with known mechanisms of microvascular remodeling. The morphological changes captured by the RAI (such as an increased arteriolar light reflex and focal narrowing) are thought to reflect arteriosclerotic processes (including hyalinization and endothelial damage) induced by chronic hemodynamic stress and hyperglycemia (rather than atherosclerotic lipid deposition, which primarily affects larger arteries) [9,10]. This mechanism is corroborated by our observation that a higher RAI is correlated with systemic biomarkers of inflammation and oxidative stress, including elevated uric acid levels and decreased serum albumin levels [17,18,19]. Furthermore, previous histological and experimental studies have confirmed that these retinal findings correspond to structural alterations observed in hypertensive and diabetic microvasculature [9,20]. Taken together, these findings suggest that the RAI may reflect the cumulative hemodynamic and metabolic burden.

The assessment of retinal vascular morphology may provide additional context in population-based screening settings. Younger adults are often underrepresented in conventional cardiovascular risk assessments, and the relatively stronger associations observed in this subgroup warrant further investigation. However, these findings should be interpreted with caution, and further studies are needed to determine whether retinal vascular changes provide incremental value beyond established risk factors. The observed sex-specific associations also merit consideration. According to the results of the stratified analyses, the association with cardiovascular disease appeared to be more pronounced in men, whereas the association with stroke appeared to be stronger in women. These differences are consistent with known sex-related variations in vascular structure and disease patterns [21,22]. Previous studies have demonstrated that compared with men, women tend to have wider retinal arterioles and experience a slower decline in arteriolar diameter until midlife [23,24]. However, a recent deep learning-based vascular imaging study suggested that this decline may become accelerated after midlife in women, thereby approaching patterns that are observed in age-matched men [25]. This transition may be related to the loss of the vasoprotective effects of estrogen during menopause. Estrogen has been observed to modulate vascular tone, inhibit smooth muscle proliferation, and reduce oxidative stress and endothelial dysfunction. Moreover, the abrupt hormonal changes occurring around the stage of menopause are known to contribute not only to retinal arteriolar remodeling but also to an increased risk of cerebrovascular stroke in women [26]. In this context, the stronger association between the RAI and stroke observed in women may reflect sex-specific differences in vascular aging and remodeling. However, further studies are needed to clarify these mechanisms. In addition to traditional vascular risk factors, we also identified associations between the RAI and laboratory biomarkers related to inflammation, oxidative stress, and metabolic dysregulation. High RAI grades were observed to be associated with elevated serum uric acid levels, as well as lower RBC, albumin and bilirubin levels. These patterns are consistent with a proinflammatory and oxidative environment. Lower RBC levels may reflect chronic inflammatory states that impair erythropoiesis, whereas elevated uric acid levels have been linked to endothelial dysfunction and vascular stiffness [17,27,28,29]. Reduced albumin and bilirubin levels may indicate diminished antioxidant capacity, which could further contribute to vascular vulnerability [18,19].

This study has several limitations. First, the cross-sectional design introduces temporal ambiguity, and causal relationships cannot be established. Second, although an association between high-grade RAI and stroke in adults younger than 40 years was observed by using Firth’s penalized regression, the small number of events resulted in wide confidence intervals, thereby requiring cautious interpretation. Third, some systemic conditions were partly based on self-reported history or medical records, which may introduce misclassification. In addition, information on stroke subtypes was not available. Fourth, multiple comparisons were not formally adjusted, and secondary analyses should be considered as being exploratory in nature. Fifth, selection bias may be present, as participants voluntarily underwent health screening. Sixth, although the intergrader reliability was excellent, retinal grading was manually performed. Finally, data on medication use (particularly antihypertensive agents that may influence vascular remodeling) were not available. Despite these limitations, the large sample size, standardized imaging protocol, and detailed clinical and laboratory data strengthen the internal validity of our findings.

In conclusion, the RAI assessed through routine fundus photography was significantly associated with systemic vascular and metabolic conditions, including cerebrovascular disease. These associations appeared to be more prominent in younger individuals and in women, thus suggesting potential age- and sex-specific vulnerabilities. Laboratory correlations with markers of inflammation and oxidative stress further support the role of the RAI as a systemic biomarker. Taken together, these findings highlight the notion that retinal vascular changes may be correlated with systemic vascular health. Longitudinal studies are needed to clarify temporal relationships and potential prognostic relevance.

## Figures and Tables

**Table 1:** Baseline Characteristics and Systemic Comorbidities of the Study Population.

Variable	Control (*n* = 71,234)	Mild RAI (*n* = 2933)	Marked RAI (*n* = 441)	*p* Value	Missing No. (%)
Age, years	48.0 (10.3)	56.4 (9.1)	58.0 (8.9)	<0.001	3 (<0.1)
Fasting glucose, mg/dL	98.0 (19.1)	105.8 (27.8)	108.6 (31.8)	<0.001	61 (0.1)
SBP, mmHg	116.9 (15.6)	128.2 (17.1)	131.8 (19.7)	<0.001	168 (0.2)
DBP, mmHg	75.8 (12.0)	82.8 (12.7)	84.1 (19.7)	<0.001	168 (0.2)
Body mass index, kg/m^2^	23.4 (3.0)	24.5 (3.0)	24.8 (3.1)	<0.001	303 (0.4)
HbA1c, %	5.7 (0.7)	6.0 (1.0)	6.1 (1.0)	<0.001	3063 (4.1)
C-reactive protein, mg/dL	0.14 (0.44)	0.19 (0.65)	0.23 (0.75)	<0.001	12,830 (17.2)
WBC, ×10^3^/μL	5.75 (1.84)	5.79 (1.67)	5.97 (1.70)	0.002	66 (0.1)
RBC, ×10^6^/μL	4.64 (0.47)	4.59 (0.47)	4.56 (0.50)	<0.001	66 (0.1)
Hemoglobin, g/dL	14.3 (1.6)	14.2 (1.7)	14.1 (1.8)	<0.001	324 (0.4)
Hematocrit, %	42.8 (4.4)	42.3 (4.4)	41.9 (4.8)	<0.001	67 (0.1)
Platelet count, ×10^3^/μL	242.4 (54.7)	241.2 (60.6)	241.8 (63.0)	0.117	324 (0.4)
Total cholesterol, mg/dL	195.2 (34.5)	198.5 (35.4)	195.9 (34.9)	<0.001	61 (0.1)
Triglyceride, mg/dL	114.0 (77.3)	125.6 (89.8)	134.6 (84.5)	<0.001	91 (0.1)
LDL-C, mg/dL	119.6 (32.1)	122.3 (32.4)	119.9 (32.5)	<0.001	1132 (1.5)
HDL-C, mg/dL	54.4 (13.4)	53.0 (13.0)	51.8 (12.9)	<0.001	87 (0.1)
Albumin, g/dL	4.38 (0.26)	4.31 (0.28)	4.30 (0.27)	<0.001	62 (0.1)
Bilirubin, mg/dL	1.08 (0.51)	1.02 (0.42)	1.00 (0.38)	<0.001	64 (0.1)
BUN, mg/dL	13.6 (3.6)	14.6 (4.6)	15.5 (7.3)	<0.001	60 (0.1)
Creatinine, mg/dL	1.00 (0.19)	1.01 (0.33)	1.05 (0.48)	0.046	63 (0.1)
Uric acid, mg/dL	5.53 (1.43)	5.84 (1.62)	5.95 (1.44)	<0.001	72 (0.1)
Calcium, mg/dL	9.23 (0.39)	9.21 (0.42)	9.23 (0.37)	0.022	261 (0.3)
	No. (%)	No. (%)	No. (%)		
**Age**				<0.001	3 (<0.1)
<40	16,774 (23.5)	92 (3.1)	6 (1.4)		
40–49	24,668 (34.6)	583 (19.9)	78 (17.7)		
50–59	19,658 (27.6)	1157 (39.4)	148 (33.6)		
60–69	8366 (11.7)	886 (30.2)	171 (38.8)		
≥70	1768 (2.5)	215 (7.3)	38 (8.6)		
**Sex**				0.220	3 (<0.1)
Male	37,909 (53.2)	1518 (51.8)	226 (51.2)		
Female	33,331 (46.8)	1415 (48.2)	215 (48.8)		
**Cigarette smoking**				<0.001	2619 (3.5)
None	36,721 (53.4)	1558 (55.6)	233 (55.0)		
Former	16,861 (24.5)	747 (26.7)	131 (30.9)		
Current	15,183 (22.1)	498 (17.8)	60 (14.2)		
**Alcohol consumption**				<0.001	3046 (4.1)
None	19,554 (28.6)	919 (32.9)	131 (31.4)		
Mild-moderate (1–4/week)	2119 (3.1)	101 (3.6)	21 (5.0)		
Severe (≥5/week)	46,678 (68.3)	1777 (63.5)	265 (63.5)		
**Physical activity**				<0.001	1271 (1.7)
Inactive (0–1/week)	24,456 (34.9)	904 (31.5)	116 (26.7)		
Moderate (≥2/week)	45,584 (65.1)	1962 (68.5)	318 (73.3)		
**Systemic comorbidities**					
Systemic HTN	16,544 (23.2)	1705 (58.1)	307 (69.8)	<0.001	16 (<0.1)
Diabetes mellitus	6637 (9.3)	551 (18.8)	117 (26.6)	<0.001	15 (<0.1)
Hyperlipidemia	16,265 (22.8)	865 (29.5)	123 (28.0)	<0.001	17 (<0.1)
Cardiovascular disease	2356 (3.3)	205 (7.0)	31 (7.1)	<0.001	115 (0.2)
Metabolic syndrome	15,601 (21.9)	1216 (41.5)	214 (48.6)	<0.001	13 (<0.1)
Cerebrovascular stroke	290 (0.4)	39 (1.3)	13 (3.0)	<0.001	341 (0.5)

Abbreviations: RAI, retinal arteriosclerosis index; N, number; SBP, systolic blood pressure; DBP, diastolic blood pressure; WBC, white blood cell; RBC, red blood cell; LDL-C, low-density lipoprotein cholesterol; HDL-C, high-density lipoprotein cholesterol; BUN, blood urea nitrogen; HTN, hypertension. Data are presented as the means (SDs) or no. (%), unless otherwise indicated. Group definitions: Control = RAI grades 0–1; Mild = grade 2; Marked = grades 3–4.

**Table 2:** Dose–response associations between Retinal Arteriosclerosis Index (RAI) grades and systemic comorbidities.

Comorbidity	RAI, Grades 0–1	RAI, Grade 2	RAI, Grades 3–4	Adjusted *p* for Trend ^‡^
	OR (95% CI)	OR (95% CI) ^†^	OR (95% CI) ^†^	
Hypertension	1 (Reference)	2.83 (2.59–3.10)	4.23 (3.31–5.40)	<0.001
Diabetes Mellitus	1 (Reference)	1.30 (1.16–1.45)	1.75 (1.37–2.25)	<0.001
Hyperlipidemia	1 (Reference)	1.02 (0.93–1.12)	0.84 (0.65–1.05)	0.541
Metabolic Syndrome	1 (Reference)	1.59 (1.45–1.74)	1.93 (1.52–2.44)	<0.001
Cardiovascular Disease	1 (Reference)	1.52 (1.29–1.79)	1.14 (0.73–1.77)	<0.001
Stroke	1 (Reference)	1.76 (1.20–2.57)	3.35 (1.74–6.45)	<0.001

Abbreviations: RAI, retinal arteriosclerosis index; OR, odds ratio; CI, confidence interval. ^†^ Models were adjusted for baseline demographic and lifestyle factors (age, sex, body mass index, smoking status, alcohol consumption, physical activity, and household income). To avoid overadjustment and circular modeling, additional clinical variables were selectively included depending on the outcome: fasting blood glucose and total cholesterol for hypertension; systolic and diastolic blood pressure and total cholesterol for diabetes mellitus; systolic and diastolic blood pressure and fasting blood glucose for hyperlipidemia; and systolic and diastolic blood pressure, fasting blood glucose, and total cholesterol for cardiovascular disease and stroke. Metabolic syndrome models were adjusted for baseline demographic and lifestyle factors only. ^‡^ Adjusted *p*-for-trend values were calculated using multivariable logistic regression models, with RAI grade treated as an ordinal variable (0 for Grades 0–1, 1 for Grade 2, and 2 for Grades 3–4).

**Table 3:** Stratified Associations of High-Grade RAI (≥2) with Systemic Comorbidities.

Sex	Men	Women	Total
	OR (95% CI)	OR (95% CI)	OR (95% CI)
Systemic HTN	2.60 (2.33–2.90)	3.47 (3.05–3.95)	2.97 (2.73–3.23)
Diabetes mellitus	1.34 (1.17–1.52)	1.38 (1.17–1.64)	1.35 (1.22–1.50)
Hyperlipidemia	0.97 (0.86–1.09)	1.02 (0.89–1.16)	0.99 (0.91–1.08)
Cardiovascular disease	1.53 (1.27–1.85)	1.31 (0.99–1.73)	1.46 (1.25–1.71)
Metabolic syndrome	1.50 (1.34–1.69)	1.79 (1.57–2.05)	1.63 (1.49–1.78)
Cerebrovascular stroke	1.34 (0.83–2.16)	3.46 (2.08–5.78)	1.98 (1.41–2.79)
**Age group**	**<40**	**40–59**	**≥60**
	OR (95% CI)	OR (95% CI)	OR (95% CI)
Systemic HTN	4.97 (3.10–7.95)	3.39 (3.05–3.77)	2.15 (1.86–2.46)
Diabetes mellitus	1.20 (0.51–2.85)	1.33 (1.16–1.53)	1.37 (1.17–1.59)
Hyperlipidemia	1.56 (0.96–2.52)	1.09 (0.98–1.21)	0.89 (0.77–1.03)
Cardiovascular disease	1.99 (0.62–6.41)	1.43 (1.13–1.81)	1.42 (1.14–1.76)
Metabolic syndrome	2.90 (1.71–4.91)	1.64 (1.46–1.83)	1.55 (1.34–1.78)
Cerebrovascular stroke	21.25 (2.22–97.14) ^†^	2.34 (1.43–3.85)	1.66 (1.03–2.68)

Abbreviations: OR, odds ratio; CI, confidence interval; HTN, hypertension. Models were adjusted for baseline demographic and lifestyle factors (age, sex, body mass index, smoking status, alcohol consumption, physical activity, and house-hold income). To avoid overadjustment and circular modeling, additional clinical variables were selectively included depending on the outcome: fasting blood glucose and total cholesterol for hypertension; systolic and diastolic blood pressure and total cholesterol for diabetes mellitus; systolic and diastolic blood pressure and fasting blood glucose for hyperlipidemia; and systolic and diastolic blood pressure, fasting blood glucose, and total cholesterol for cardiovascular disease and stroke. Metabolic syndrome models were adjusted for baseline demographic and lifestyle factors only. † For this subgroup, the odds ratio was estimated using Firth’s penalized logistic regression. To minimize overfitting, the model was adjusted using a parsimonious set of covariates, including sex, body mass index, and smoking status.

## Data Availability

The data that support the findings of this study are available from the corresponding author upon reasonable request.

## Abbreviations

RAI	retinal arteriosclerosis index
AV	arteriovenous
SBP	systolic blood pressure
DBP	diastolic blood pressure
BMI	body mass index
HTN	hypertension
DM	diabetes mellitus
LDL-C	low-density lipoprotein cholesterol
HDL-C	high-density lipoprotein cholesterol
BUN	blood urea nitrogen
WBC	white blood cell
RBC	red blood cell

## References

[1] Luyten L.J., Dockx Y., Madhloum N., Sleurs H., Gerrits N., Janssen B.G., Neven K.Y., Plusquin M., Provost E.B., De Boever P. (2020). Association of Retinal Microvascular Characteristics With Short-term Memory Performance in Children Aged 4 to 5 Years. JAMA Netw. Open.

[2] Kawasaki R., Wang J.J., Rochtchina E., Taylor B., Wong T.Y., Tominaga M., Kato T., Daimon M., Oizumi T., Kawata S. (2006). Cardiovascular risk factors and retinal microvascular signs in an adult Japanese population: The Funagata Study. Ophthalmology.

[3] Wong T.Y., Klein R., Klein B.E., Tielsch J.M., Hubbard L., Nieto F.J. (2001). Retinal microvascular abnormalities and their relationship with hypertension, cardiovascular disease, and mortality. Surv. Ophthalmol..

[4] Lusk J.B., Nalawade V., Wilson L.E., Song A., Schrag M., Biousse V., Dumitrascu O., Poli S., Piccini J., Hammill B. (2025). Atrial Fibrillation and Retinal Stroke. JAMA Netw. Open.

[5] Hwang D.D., Lee K.E., Kim Y., Kim M.S., Rim T.H., Kim M., Kim H., Kyoung D.S., Park J.I. (2023). Incidence of Retinal Artery Occlusion and Related Mortality in Korea, 2005 to 2018. JAMA Netw. Open.

[6] Brinchmann-Hansen O., Sandvik L. (1986). The intensity of the light reflex on retinal arteries and veins. Acta Ophthalmol..

[7] Kaushik S., Tan A.G., Mitchell P., Wang J.J. (2007). Prevalence and associations of enhanced retinal arteriolar light reflex: A new look at an old sign. Ophthalmology.

[8] Scheie H.G. (1953). Evaluation of ophthalmoscopic changes of hypertension and arteriolar sclerosis. AMA Arch. Ophthalmol..

[9] Wong T.Y., Mitchell P. (2004). Hypertensive retinopathy. N. Engl. J. Med..

[10] Garner A., Ashton N., Tripathi R., Kohner E.M., Bulpitt C.J., Dollery C.T. (1975). Pathogenesis of hypertensive retinopathy. An experimental study in the monkey. Br. J. Ophthalmol..

[11] Wong T.Y., Klein R., Couper D.J., Cooper L.S., Shahar E., Hubbard L.D., Wofford M.R., Sharrett A.R. (2001). Retinal microvascular abnormalities and incident stroke: The Atherosclerosis Risk in Communities Study. Lancet.

[12] Lee C., Choe E.K., Choi J.M., Hwang Y., Lee Y., Park B., Chung S.J., Kwak M.S., Lee J.E., Kim J.S. (2018). Health and Prevention Enhancement (H-PEACE): A retrospective, population-based cohort study conducted at the Seoul National University Hospital Gangnam Center, Korea. BMJ Open.

[13] Rim T.H., Lee G., Kim Y., Tham Y.C., Lee C.J., Baik S.J., Kim Y.A., Yu M., Deshmukh M., Lee B.K. (2020). Prediction of systemic biomarkers from retinal photographs: Development and validation of deep-learning algorithms. Lancet Digit. Health.

[14] Ma D.J., Oh B.L., Bak E., Kim J.S., Lee J., Choi H.J. (2024). A Comprehensive Health Screening Program Reveals the Prevalence of and Risk Factors for Age-Related Macular Degeneration: A Cross-Sectional Analysis. Biomedicines.

[15] Choe S., Kim Y.K., Park K.H., Choi H.J., Jeoung J.W. (2023). Natural History of Optic Disc With Physiologic Large Cup: Incidence, Predictors of Glaucoma Conversion After Minimum 10-Year Follow-up. Am. J. Ophthalmol..

[16] Tan C.E., Ma S., Wai D., Chew S.K., Tai E.S. (2004). Can we apply the National Cholesterol Education Program Adult Treatment Panel definition of the metabolic syndrome to Asians?. Diabetes Care.

[17] Hayfron-Benjamin C.F., van den Born B.J., Amoah A.G.B., Maitland-van der Zee A.H., Meeks K.A.C., Beune E., Klipstein-Grobusch K., Agyemang C. (2021). Associations of Serum Uric Acid Levels With Macrovascular and Renal Microvascular Dysfunction Among Individuals From Sub-Saharan Africa. JAMA Netw. Open.

[18] Zhu L., Chen M., Lin X. (2020). Serum albumin level for prediction of all-cause mortality in acute coronary syndrome patients: A meta-analysis. Biosci. Rep..

[19] Ziberna L., Jenko-Praznikar Z., Petelin A. (2021). Serum Bilirubin Levels in Overweight and Obese Individuals: The Importance of Anti-Inflammatory and Antioxidant Responses. Antioxidants.

[20] Nag T.C., Maurya M., Roy T.S. (2019). Age-related changes of the human retinal vessels: Possible involvement of lipid peroxidation. Ann. Anat..

[21] Ji H., Kwan A.C., Chen M.T., Ouyang D., Ebinger J.E., Bell S.P., Niiranen T.J., Bello N.A., Cheng S. (2022). Sex Differences in Myocardial and Vascular Aging. Circ. Res..

[22] Bots S.H., Peters S.A.E., Woodward M. (2017). Sex differences in coronary heart disease and stroke mortality: A global assessment of the effect of ageing between 1980 and 2010. BMJ Glob. Health.

[23] Cheung C.Y., Tay W.T., Mitchell P., Wang J.J., Hsu W., Lee M.L., Lau Q.P., Zhu A.L., Klein R., Saw S.M. (2011). Quantitative and qualitative retinal microvascular characteristics and blood pressure. J. Hypertens..

[24] Ponto K.A., Werner D.J., Wiedemer L., Laubert-Reh D., Schuster A.K., Nickels S., Hohn R., Schulz A., Binder H., Beutel M. (2017). Retinal vessel metrics: Normative data and their use in systemic hypertension: Results from the Gutenberg Health Study. J. Hypertens..

[25] Mitamura M., Saito M., Fukutsu K., Dong Z., Ando R., Kase S., Noda K., Shiba R., Isogai N., Dohke M. (2025). Sex Differences in Age-Related Changes in Retinal Arteriovenous Area Based on Deep Learning Segmentation Model. Ophthalmol. Sci..

[26] Bushnell C.D., Kapral M.K. (2023). Stroke in Women and Unique Risk Factors. Stroke.

[27] Colin Y., Le Van Kim C., El Nemer W. (2014). Red cell adhesion in human diseases. Curr. Opin. Hematol..

[28] Byrnes J.R., Wolberg A.S. (2017). Red blood cells in thrombosis. Blood.

[29] Hao M., Jiang S., Tang J., Li X., Wang S., Li Y., Wu J., Hu Z., Zhang H. (2024). Ratio of Red Blood Cell Distribution Width to Albumin Level and Risk of Mortality. JAMA Netw. Open.

